# Editorial: Recent Trends in Optical and Mechanical Characterization of Nanomaterials

**DOI:** 10.3389/fchem.2020.564014

**Published:** 2020-10-02

**Authors:** Stefan G. Stanciu, Loredana Latterini, Costas A. Charitidis

**Affiliations:** ^1^Center for Microscopy-Microanalysis and Information Processing, Politehnica University of Bucharest, Bucharest, Romania; ^2^Department of Chemistry, Biology and Biotechnology, University of Perugia, Perugia, Italy; ^3^RNANO Lab—Research Unit of Advanced, Composite, Nano Materials & Nanotechnology, School of Chemical Engineering, National Technical University of Athens, Athens, Greece

**Keywords:** nanomaterials, optical characterization, mechanical characterization, nanoparticles, 2D materials

Over the course of the past few years we have witnessed a series of paradigm shifts in many critical fields of science driven by the development of novel nanomaterials. At present, applications of these nanostructured or nanosized advanced materials impact almost all aspects of modern life. One of the critical fields that have been tremendously impacted by nanomaterials is medicine. Among the wide range of biomedical applications that are augmented by the ingenious use of novel nanosized materials, we feel important to mention nanomaterial-based Point-of-Care (POC) devices that can provide a quick screening or diagnostics test, in an affordable way, next to the patient, including in low-resource settings. The reason for highlighting this specific application relates to the current global health emergency (Sohrabi et al., 2020) taking place at the time of writing this Editorial, when all eyes are on the SARS-CoV-2 virus (Zhu et al., 2020) pandemic. In less than 6 months since the first diagnosed case in China, we have witnessed a very swift spread resulting in millions of confirmed cases and hundreds of thousands of deaths. These numbers have to do with the limited availability of tests (hence low testing rate), given that testing represents the key to addressing any epidemy, so that infected subjects can be identified and isolated. As the number of SARS-CoV-2 cases exploded in very short time, the demand for fast and precise testing tools skyrocketed, but this surge could not be handled by means of traditional tests, e.g., real-time reverse-transcription polymerase chain reaction (RT-PCR) (Corman et al., 2012), so alternative solutions had to be rapidly identified (Sheridan, 2020). A question that naturally arises in the context of this Research Topic is the following: could nanomaterials be of help in such situations, so that in the future they will provide reliable and accessible solutions capable to face potential similar problems more efficiently? Recent progress in nanomaterial-based POC devices surely suggest so (Ge et al., 2014; Wang et al., 2016; Hu et al., 2017; Quesada-González and Merkoçi, 2018; Xia et al., 2019). Further efforts in this direction will surely soon result in very efficient, affordable, and easily accessible solutions to diagnose infectious (but also non-communicable) diseases. Important side effects of this challenging COVID-19 crisis such as reduced transportation or industrial activities, with lockdowns instituted in many countries across all continents, were found to reflect in our surrounding environment (Aloi et al., 2020; Isaifan, 2020). We have witnessed bluer skies, fresher air and cleaner water, features that we certainly do not want to lose once this pandemic will end, and things return to normal. We can raise once more the same question, could nanomaterials be of help? The answer is again “Yes,” and for this we want to recall that massive progress has been reported lately in the field of nanomaterials that can adsorb or degrade pollutants (Khin et al., 2012; Gong et al., 2018; Lu and Astruc, 2018, 2020; Wen et al., 2019; Wu et al., 2019). Extending these efforts will, without doubt, contribute significantly to the sustainability of our surrounding environment. Green energy is a closely related field that also greatly benefits of key advances in nanomaterials (Fan et al., 2016; Sun et al., 2017; Abdalla et al., 2018; Ullattil et al., 2018; Arunkumar et al., 2019). Endeavors addressing this critical domain will surely help sidelining fossil fuels not far from now, which is a tremendously important objective if considering that greenhouse gas emissions are among the major contributors to the global warming phenomena (Lashof and Ahuja, 1990; McGlade and Ekins, 2015).

All these scientific and technological enterprises focused on harnessing the immense potential of nanomaterials to completely revolutionize critical fields and improve our life would not be possible without the availability of a wide range of investigation techniques, methods, and protocols. These allow the in-depth characterization of nanomaterials and related processes at sufficient spatial and temporal resolution to accurately understand their properties and characteristics. Such thorough understanding is fundamental for improving synthesis protocols, for discovering hidden potential or faults, for predicting behaviors and for many other equally important tasks. Needless to say, the number of physico-chemical properties that require to be probed and characterized for achieving in-depth knowledge of any emerging advanced material is huge. However, probably not many will contradict that the optical and mechanical properties are some of the most important to consider (Tan and Lim, 2006; Zhang, 2009; Guo et al., 2013; Bauer et al., 2017; Papageorgiou et al., 2017). Such properties can account for the different ways by which a nanomaterial can: interact with a biological structure (Anselmo et al., 2015; Yao et al., 2020), enable functional scaffolds (Castro et al., 2015; Zhang et al., 2018), report a diseases marker (Huang et al., 2007), provide contrast in bioimaging (West and Halas, 2003; Le Trequesser et al., 2013), deliver drugs (Rwei et al., 2015; Yang et al., 2016), signal an environmental condition (Mauter and Elimelech, 2008; Su et al., 2012; Maduraiveeran and Jin, 2017), harvest energy (Wang, 2012; Fan et al., 2016; Ishii et al., 2019), enforce security (Arppe and Sørensen, 2017; Liu et al., 2019; Ko et al., 2020) etc. Noteworthy, the optical and mechanical characteristics of nanomaterials are bound in many ways, and investigating them in-tandem with complementary techniques addressing both parts can reveal significant aspects that enable better functionalization strategies and enhanced applications (Gilroy et al., 2016; Zheludev and Plum, 2016; Ling et al., 2018).

The Frontiers in Chemistry Research Topic entitled “*Recent Trends in Optical and Mechanical Characterization of Nanomaterials”* is comprised of four contributions presenting both original research and one review, touching hot subjects that can promote the further translation of latest generation nanomaterials to pioneering nanotechnologies. In the Review article of Kröner and Hirsch the authors discuss different characterization methods for two-dimensional carbon-based materials, ranging from light microscopy, scanning electron microscopy, transmission electron microscopy, scanning transmission electron microscopy, scanning tunneling microscopy (conductive), atomic force microscopy, scanning electrochemical microscopy, Raman spectroscopy, UV–vis, X-ray photoelectron spectroscopy, X-ray fluorescence spectroscopy, energy-dispersive X-ray spectroscopy, Auger electron spectroscopy, electron energy loss spectroscopy, X-ray diffraction, inductively coupled plasma atomic emission spectroscopy to dynamic light scattering. They highlight how these methods are of utmost importance for resolving fundamental properties of the 2D materials, such as their size and shape, layer structure, conductivity, defects, chemical composition, and others. The final choice on what method to apply depends on the nanomaterial sample and the specific scenario that researcher deals with. Switching our attention to original research efforts, in the interesting experiment presented by Bonatti et al., a recent fully atomistic model, ωFQ, that relies on the Drude theory, electrostatics, and quantum tunneling concepts, is employed for calculating the optical properties of complex Na, Ag, and Au nanostructures characterized by different geometrical arrangements. The results achieved by means of ωFQ are compared with those that can be obtained using continuum Boundary Element Method (BEM) calculations, and an insightful discussion on theoretical analogies and differences between the two alternatives is carried out, highlighting the physical quantities underlying both approaches. One of the most important conclusions is that the main difference between ωFQ and BEM is that the ωFQ approach retains the atomistic nature of nanoparticles, while BEM models such nanomaterials only in terms of their surface. Also addressing noble-metal nanomaterials, Gambucci et al., present a new method for preparing free-standing keratin-based films containing Au/Ag nanorods. A special focus is placed on assessing how the surface chemistry of the considered nanostructures influences the optical and mechanical properties of the developed keratin composite films, also highlighting interdependencies that exist between these characteristics. In this extensive analysis, they present important findings such that the Ag/Au nanorods: (i) confer to the keratin films interesting color effects as a result of the plasmonic absorptions (ii) significantly modify keratin's fluorescence emission, (iii) favor the presence of more packed supramolecular structures in the films, or (iv) induce an increase in the Young's modulus of the keratin thin films. These results show that by tuning one or more properties of the Ag/Au nanorods, a wide range of simultaneous effects in terms of the keratin thin film's characteristics take place. Also focusing on the fluorescence of nanomaterials and related properties, Genovese et al. introduce with their valuable work a new type of multifluorophoric silica nanoparticles which they design to efficiently absorb the Cerenkov radiation (CR), a type of electromagnetic radiations whose utility for enabling *in-vivo* imaging is gaining increasing attention. The proposed contrast agents are doped with five different dyes and can efficiently convert part of the CR into near-infrared fluorescence, improving tissue penetration, one of the most important bottlenecks of Cerenkov luminescence imaging (CLI). Given that the brightness of the proposed NPs is remarkably high with many different excitation/emission filter setups they are compatible with a wide variety of CLI configurations that differ in terms of illumination lines. The authors show that their proposed nanomaterial can enable imaging of deep tissue features, demonstrating tissue detection exceeding 1.0-cm depths, pushing forward the state-of-the art on CLI.

This collection of articles demonstrates once more the importance of employing modern tools and approaches to characterize nanomaterials from various optical and mechanical perspectives. By understanding in high-depth their properties, new breakthrough applications that augment the current state-of-the art in critical fields of science and technology can be achieved. In this context, we find noteworthy to mention that one of the great opportunities in nanomaterials research is artificial intelligence (AI) (LeCun et al., 2015). Synergistically combining characterization techniques with AI methods will result in unprecedented possibilities, unavailable with experimental-only approaches, as emerging work on the topic demonstrates (Goh et al., 2017; Wang et al., 2019). Bridging the communities of materials and AI scientists will be one of the main important challenges to achieve new paradigms and approaches in nanomaterials characterization that can give rise to an exponential growth of novel applications and hugely enhance current ones.

## Author Contributions

SS, LL, and CC wrote and reviewed this manuscript. All authors contributed to the article and approved the submitted version.

## Conflict of Interest

The authors declare that the research was conducted in the absence of any commercial or financial relationships that could be construed as a potential conflict of interest.

## References

[B1] Abdalla A. M. Hossain S. Azad A. T. Petra P. M. I. Begum F. Eriksson S. G. (2018). Nanomaterials for solid oxide fuel cells: a review. Renew. Sustain. Energy Rev. 82, 353–368. 10.1016/j.rser.2017.09.046

[B2] Aloi A. Alonso B. Benavente J. Cordera R. Echániz E. González F. (2020). Effects of the COVID-19 lockdown on urban mobility: empirical evidence from the City of Santander (Spain). Sustainability 12:3870 10.3390/su12093870

[B3] Anselmo A. C. Zhang M. Kumar S. Vogus D. R. Menegatti S. Helgeson M. E.. (2015). Elasticity of nanoparticles influences their blood circulation, phagocytosis, endocytosis, and targeting. ACS Nano 9, 3169–3177. 10.1021/acsnano.5b00147 25715979

[B4] Arppe R. Sørensen T. J. (2017). Physical unclonable functions generated through chemical methods for anti-counterfeiting. Nat. Rev. Chem. 1:0031 10.1038/s41570-017-0031

[B5] Arunkumar T. Ao Y. Luo Z. Zhang L. Li J. Denkenberger D. (2019). Energy efficient materials for solar water distillation-A review. Renew. Sustain. Energy Rev. 115:109409 10.1016/j.rser.2019.109409

[B6] Bauer J. Meza L. R. Schaedler T. A. Schwaiger R. Zheng X. Valdevit L. (2017). Nanolattices: an emerging class of mechanical metamaterials. Adv. Mater. 29:1701850. 10.1002/adma.201701850 28873250

[B7] Castro N. J. O'brien J. Zhang L. G. (2015). Integrating biologically inspired nanomaterials and table-top stereolithography for 3D printed biomimetic osteochondral scaffolds. Nanoscale 7:14010–14022. 10.1039/C5NR03425F 26234364 PMC4537413

[B8] Corman V. Eckerle I. Bleicker T. Zaki A. Landt O. Eschbach-Bludau M. (2012). Detection of a novel human coronavirus by real-time reverse-transcription polymerase chain reaction. Euro Surveill. 17:20285 10.2807/ese.17.39.20285-en 23041020

[B9] Fan F. R. Tang W. Wang Z. L. (2016). Flexible nanogenerators for energy harvesting and self-powered electronics. Adv. Mater. 28, 4283–4305. 10.1002/adma.201504299 26748684

[B10] Ge X. Asiri A. M. Du D. Wen W. Wang S. Lin Y. (2014). Nanomaterial-enhanced paper-based biosensors. TrAC Trends Anal. Chem. 58, 31–39. 10.1016/j.trac.2014.03.008

[B11] Gilroy K. D. Ruditskiy A. Peng H.-C. Qin D. Xia Y. (2016). Bimetallic nanocrystals: syntheses, properties, and applications. Chem. Rev. 116, 10414–10472. 10.1021/acs.chemrev.6b00211 27367000

[B12] Goh G. B. Hodas N. O. Vishnu A. (2017). Deep learning for computational chemistry. J. Comput. Chem. 38, 1291–1307. 10.1002/jcc.24764 28272810

[B13] Gong X. Huang D. Liu Y. Peng Z. Zeng G. Xu P.. (2018). Remediation of contaminated soils by biotechnology with nanomaterials: bio-behavior, applications, and perspectives. Crit. Rev. Biotechnol. 38, 455–468. 10.1080/07388551.2017.1368446 28903604

[B14] Guo D. Xie G. Luo J. (2013). Mechanical properties of nanoparticles: basics and applications. J. Phys. D Appl. Phys. 47:013001 10.1088/0022-3727/47/1/013001

[B15] Hu J. Jiang Y.- Z. Wu L.- L. Wu Z. Bi Y. Wong G.. (2017). Dual-signal readout nanospheres for rapid point-of-care detection of ebola virus glycoprotein. Anal. Chem. 89, 13105–13111. 10.1021/acs.analchem.7b02222 29148720

[B16] Huang X. Jain P. K. El-Sayed I. H. El-Sayed M. A. (2007). Gold nanoparticles: interesting optical properties and recent applications in cancer diagnostics and therapy. Nanomedicine 2, 681–693. 10.2217/17435889.2.5.681 17976030

[B17] Isaifan R. (2020). The dramatic impact of Coronavirus outbreak on air quality: has it saved as much as it has killed so far? Global J. Environ. Sci. Manage. 6, 275–288. 10.22034/GJESM.2020.03.01

[B18] Ishii S. Shinde S. L. Nagao T. (2019). Nonmetallic materials for plasmonic hot carrier excitation. Adv. Opt. Mater. 7:1800603 10.1002/adom.201800603

[B19] Khin M. M. Nair A. S. Babu V. J. Murugan R. Ramakrishna S. (2012). A review on nanomaterials for environmental remediation. Energy Environ. Sci. 5, 8075–8109. 10.1039/c2ee21818f

[B20] Ko J. H. Yoo Y. J. Kim Y. J. Lee S. S. Song Y. M. (2020). Flexible, large-area covert polarization display based on ultrathin lossy nanocolumns on a metal film. Adv. Funct. Mater. 30:1908592 10.1002/adfm.201908592

[B21] Lashof D. A. Ahuja D. R. (1990). Relative contributions of greenhouse gas emissions to global warming. Nature 344, 529–531. 10.1038/344529a0

[B22] Le Trequesser Q. Seznec H. Delville M.- H. (2013). Functionalized nanomaterials: their use as contrast agents in bioimaging: mono-and multimodal approaches. Nanotechnol. Rev. 2, 125–169. 10.1515/ntrev-2012-0080

[B23] LeCun Y. Bengio Y. Hinton G. (2015). Deep learning. Nature 521:436. 10.1038/nature14539 26017442

[B24] Ling S. Kaplan D. L. Buehler M. J. (2018). Nanofibrils in nature and materials engineering. Nat. Rev. Mater. 3, 1–15. 10.1038/natrevmats.2018.16 34168896 PMC8221570

[B25] Liu Y. Han F. Li F. Zhao Y. Chen M. Xu Z.. (2019). Inkjet-printed unclonable quantum dot fluorescent anti-counterfeiting labels with artificial intelligence authentication. Nat. Commun. 10:2409. 10.1038/s41467-019-10406-7 31160579 PMC6547729

[B26] Lu F. Astruc D. (2018). Nanomaterials for removal of toxic elements from water. Coord. Chem. Rev. 356, 147–164. 10.1016/j.ccr.2017.11.003

[B27] Lu F. Astruc D. (2020). Nanocatalysts and other nanomaterials for water remediation from organic pollutants. Coord. Chem. Rev. 408:213180 10.1016/j.ccr.2020.213180

[B28] Maduraiveeran G. Jin W. (2017). Nanomaterials based electrochemical sensor and biosensor platforms for environmental applications. Trends Environ. Anal. Chem. 13, 10–23. 10.1016/j.teac.2017.02.001

[B29] Mauter M. S. Elimelech M. (2008). Environmental applications of carbon-based nanomaterials. Environ. Sci. Technol. 42, 5843–5859. 10.1021/es8006904 18767635

[B30] McGlade C. Ekins P. (2015). The geographical distribution of fossil fuels unused when limiting global warming to 2 C. Nature 517, 187–190. 10.1038/nature14016 25567285

[B31] Papageorgiou D. G. Kinloch I. A. Young R. J. (2017). Mechanical properties of graphene and graphene-based nanocomposites. Prog. Mater. Sci. 90, 75–127. 10.1016/j.pmatsci.2017.07.004

[B32] Quesada-González D. Merkoçi A. (2018). Nanomaterial-based devices for point-of-care diagnostic applications. Chem. Soc. Rev. 47, 4697–4709. 10.1039/C7CS00837F 29770813

[B33] Rwei A. Y. Wang W. Kohane D. S. (2015). Photoresponsive nanoparticles for drug delivery. Nano Today 10, 451–467. 10.1016/j.nantod.2015.06.004 26644797 PMC4669578

[B34] Sheridan C. (2020). Fast, portable tests come online to curb coronavirus pandemic. Nat. Biotechnol. 38, 515–518. 10.1038/d41587-020-00010-2 32203294

[B35] Sohrabi C. Alsafi Z. O'Neill N. Khan M. Kerwan A. Al-Jabir A.. (2020). World Health Organization declares global emergency: A review of the 2019 novel coronavirus (COVID-19). Int. J. Surg. 76, 71–76. 10.1016/j.ijsu.2020.02.034 32112977 PMC7105032

[B36] Su S. Wu W. Gao J. Lu J. Fan C. (2012). Nanomaterials-based sensors for applications in environmental monitoring. J. Mater. Chem. 22, 18101–18110. 10.1039/c2jm33284a

[B37] Sun H. Zhang Y. Zhang J. Sun X. Peng H. (2017). Energy harvesting and storage in 1D devices. Nat. Rev. Mater. 2, 1–12. 10.1038/natrevmats.2017.23

[B38] Tan E. Lim C. (2006). Mechanical characterization of nanofibers–a review. Compos. Sci. Technol. 66, 1102–1111. 10.1016/j.compscitech.2005.10.003

[B39] Ullattil S. G. Narendranath S. B. Pillai S. C. Periyat P. (2018). Black TiO2 nanomaterials: a review of recent advances. Chem. Eng. J. 343, 708–736. 10.1016/j.cej.2018.01.069

[B40] Wang H. Sugiarto S. Li T. Ang W. H. Lee C. Pastorin G. (2016). Advances in nanomaterials and their applications in point of care (POC) devices for the diagnosis of infectious diseases. Biotechnol. Adv. 34, 1275–1288. 10.1016/j.biotechadv.2016.09.003 27686397 PMC7127209

[B41] Wang X. (2012). Piezoelectric nanogenerators—Harvesting ambient mechanical energy at the nanometer scale. Nano Energy 1, 13–24. 10.1016/j.nanoen.2011.09.001

[B42] Wang M. Wang T. Cai P. Chen X. (2019). Nanomaterials discovery and design through machine learning. Small Methods 3:1900025 10.1002/smtd.201900025

[B43] Wen M. Li G. Liu H. Chen J. An T. Yamashita H. (2019). Metal–organic framework-based nanomaterials for adsorption and photocatalytic degradation of gaseous pollutants: recent progress and challenges. Environ. Sci. Nano 6, 1006–1025. 10.1039/C8EN01167B

[B44] West J. L. Halas N. J. (2003). Engineered nanomaterials for biophotonics applications: improving sensing, imaging, and therapeutics. Annu. Rev. Biomed. Eng. 5, 285–292. 10.1146/annurev.bioeng.5.011303.120723 14527314

[B45] Wu Y. Pang H. Liu Y. Wang X. Yu S. Fu D.. (2019). Environmental remediation of heavy metal ions by novel-nanomaterials: a review. Environ. Pollut. 246, 608–620. 10.1016/j.envpol.2018.12.076 30605816

[B46] Xia Y. Chen Y. Tang Y. Cheng G. Yu X. He H.. (2019). Smartphone-based point-of-care microfluidic platform fabricated with a ZnO nanorod template for colorimetric virus detection. ACS sensors 4, 3298–3307. 10.1021/acssensors.9b01927 31769284

[B47] Yang K. Feng L. Liu Z. (2016). Stimuli responsive drug delivery systems based on nano-graphene for cancer therapy. Adv. Drug Deliv. Rev. 105, 228–241. 10.1016/j.addr.2016.05.015 27233212

[B48] Yao C. Akakuru O. U. Stanciu S. G. Hampp N. Jin Y. Zheng J.. (2020). Effect of elasticity on the phagocytosis of micro/nanoparticles. J. Mater. Chem. B 8, 2381–2392. 10.1039/C9TB02902H 32100802

[B49] Zhang Z. Klausen L. H. Chen M. Dong M. (2018). Electroactive scaffolds for neurogenesis and myogenesis: graphene-based nanomaterials. Small 14:1801983. 10.1002/smll.201801983 30264534

[B50] Zhang Z. J. (2009). Optical Properties and Spectroscopy of Nanomaterials. Singapore: World Scientific.

[B51] Zheludev N. I. Plum E. (2016). Reconfigurable nanomechanical photonic metamaterials. Nat. Nanotechnol. 11:16. 10.1038/nnano.2015.302 26740040

[B52] Zhu N. Zhang D. Wang W. Li X. Yang B. Song J.. (2020). A novel coronavirus from patients with pneumonia in China, 2019. N. Engl. J. Med. 382, 727–733. 10.1056/NEJMoa2001017 31978945 PMC7092803

